# In Vitro Effects of Methylprednisolone over Oligodendroglial Cells: Foresight to Future Cell Therapies

**DOI:** 10.3390/cells12111515

**Published:** 2023-05-30

**Authors:** Ulises Gómez-Pinedo, Jordi A. Matías-Guiu, Denise Ojeda-Hernandez, Sarah de la Fuente-Martin, Ola Mohamed-Fathy Kamal, Maria Soledad Benito-Martin, Belen Selma-Calvo, Paloma Montero-Escribano, Jorge Matías-Guiu

**Affiliations:** 1 Laboratory of Neurobiology, Institute of Neurosciences, IdISSC, Hospital Clínico San Carlos, Universidad Complutense de Madrid, 28040 Madrid, Spain; doddydenise@gmail.com (D.O.-H.); sdelafuentemartin@gmail.com (S.d.l.F.-M.); aolakamal@gmail.com (O.M.-F.K.); msbm65@gmail.com (M.S.B.-M.); belselma@ucm.es (B.S.-C.); matiasguiu@gmail.com (J.M.-G.); 2 Department of Neurology, Institute of Neurosciences, IdISSC, Hospital Clínico San Carlos, Universidad Complutense de Madrid, 28040 Madrid, Spain; jordimatiasguiu@hotmail.com (J.A.M.-G.); pmontero84@gmail.com (P.M.-E.)

**Keywords:** multiple sclerosis, HOG cells, oligodendrocyte precursor cells, oligodendrocytes, corticosteroids, demyelination, remyelination

## Abstract

The implantation of oligodendrocyte precursor cells may be a useful therapeutic strategy for targeting remyelination. However, it is yet to be established how these cells behave after implantation and whether they retain the capacity to proliferate or differentiate into myelin-forming oligodendrocytes. One essential issue is the creation of administration protocols and determining which factors need to be well established. There is controversy around whether these cells may be implanted simultaneously with corticosteroid treatment, which is widely used in many clinical situations. This study assesses the influence of corticosteroids on the capacity for proliferation and differentiation and the survival of human oligodendroglioma cells. Our findings show that corticosteroids reduce the capacity of these cells to proliferate and to differentiate into oligodendrocytes and decrease cell survival. Thus, their effect does not favour remyelination; this is consistent with the results of studies with rodent cells. In conclusion, protocols for the administration of oligodendrocyte lineage cells with the aim of repopulating oligodendroglial niches or repairing demyelinated axons should not include corticosteroids, given the evidence that the effects of these drugs may undermine the objectives of cell transplantation.

## 1. Introduction

Various lines of research have suggested that the implantation of oligodendrocyte lineage cells able to differentiate into myelin-forming oligodendrocytes may be a useful strategy [1,2,3,4,5,6] in promoting myelin repair after a white matter lesion [7], for example, in multiple sclerosis (MS) or in case of stroke [8]. Following myelin damage, oligodendrocyte precursor cells (OPCs) migrate to the lesion site and participate in remyelination to repair the damage [9,10,11]. To this end, OPCs proliferate and subsequently differentiate into mature oligodendrocytes with the capacity to restore damaged myelin [12]; deficient remyelination is considered to be one of the main factors contributing to neurological disease progression [13]. The transplantation of oligodendrocyte lineage cells may favour remyelination [14,15,16,17,18,19], thus representing a promising strategy for the repair of central nervous system lesions [20]. Remyelination capacity is influenced by numerous factors, including age [21,22], as well as the involvement of an inflammatory component in lesions and the use of various drugs, including corticosteroids.

This study aims to determine the influence of corticosteroids on the capacity of OPCs to proliferate and differentiate into oligodendrocytes, which is essential to the future development of protocols for the transplantation of these cells to treat myelin disorders such as MS. To analyse the effect of corticosteroids, we used the HOG cell line, which is often used in vitro to analyse oligodendrocyte differentiation [23,24,25,26,27] and in models of demyelination [28].

## 2. Materials and Methods

**Figure 1:**
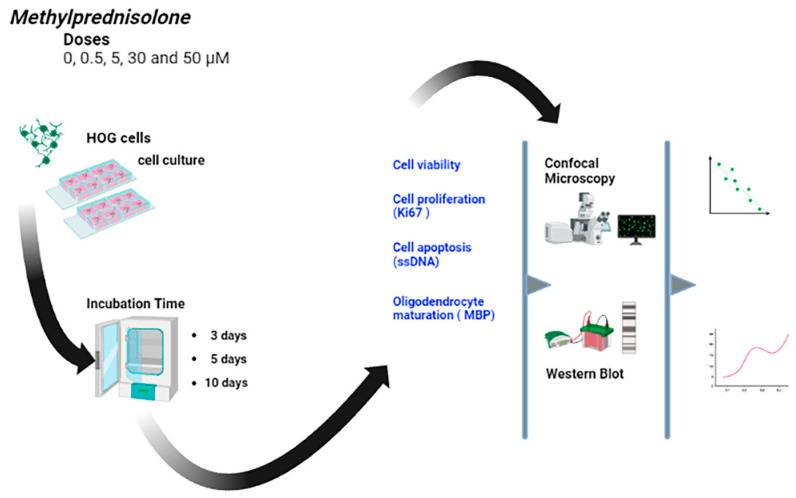
Graphical summary of the study procedure, with the 4 doses of methylprednisolone used in the cultures of HOG cells, the different incubation times, and the variables analysed.

Figure 1 shows a graphical summary of the study design. We used cultures of HOG cells treated with different doses of methylprednisolone and analysed the proliferation and differentiation of these cells at different incubation times.

### 2.1. HOG Cell Culture

This cell line is derived from a human oligodendroglioma (HOG cell line, catalogue No. SCC163; Sigma-Aldrich, St. Louis, MO, USA) and was cultured in Dulbecco’s Modified Eagle’s Medium (DMEM 1×; glutamine 2 mM, 10% foetal bovine serum, and 1% penicillin-streptomycin antibiotic) at 37 °C, in darkness, at 5% CO_2_ and 95% relative humidity. We sewed 1.5 × 10^5^ cells in 4-well chamber slides (Eppendorf, Hamburg, Germany, ref. 30742060) and incubated them for 24 h in DMEM 1× (supplemented with 10% foetal bovine serum and 1% antibiotic-antimycotic) to adhere and metabolically activate the cells (eighth passage). DMEM is considered as a maintenance culture medium, not for differentiation, since it is not added by hormones or factors that induce oligodendroglial maturation. All experiments were performed in triplicate for each of the variables used. After 24 h, the medium was changed for another containing methylprednisolone at different doses (0.5, 5, 30, or 50 μM). The calculations were carried out following the maximum recommended dosage for patients with acute flare-ups with multiple sclerosis. An amount of 1 gr of methylprednisolone is considered the limit of therapeutic dose used, which would be 50 µM of methylprednisolone in culture; 30 µM would be the corresponding dose of 600 mg; 5 µM would be the corresponding dose of 100 mg; and 0.5 µM would be the equivalent of 10 mg, approximately.

Technical data on the reagents and materials used are included in Supplementary Material Table S1.

At 3, 5, and 10 days of incubation, we analysed cell viability using a LUNA-II automated cell counter (Isogen™) system. Cells were counterstained with methylene blue at 0.5% in 0.1 M phosphate-buffered saline (PBS) for subsequent analysis of viability. All cultures were performed in four replicates.

### 2.2. Analysis of HOG Cell Proliferation and Maturation

Immunocytochemistry testing of the incubated cells was conducted to quantify the ratios of cell proliferation and maturation. Cultures were fixed with 4% paraformaldehyde in 0.1 M PBS at a pH of 7.4. They were stored at 4 °C in lead azide (Pb 0.1 M, sodium azide 0.1%, and Milli-Q water) until immunocytochemistry testing, at which time they were treated with primary antibodies labelling Ki67 (Abcam, Cambridge, UK, catalogue No. ab16667), a protein expressed during cell division; MBP (Abcam, catalogue No. AB65988), a protein present in oligodendroglial cells with myelination capacity; and ssDNA (Millipore MAB3299), which provides a cellular marker specific for apoptotic death that is independent of internucleosomal DNA fragmentation and is useful for the detection of different stages of apoptosis. Cultures were incubated overnight with the primary antibodies at 4 °C. The following day, they were washed with a solution of PBS, triton, and albumin, and incubated with secondary antibodies (goat anti-rabbit IgG, Alexa Fluor 488 or 555) for 2 h at room temperature, in darkness, counterstaining nuclei with DAPI (DAPI 1:3000, 422801, BioLegend, San Diego, CA, USA) and mounted in FluorSave™ mounting medium (catalogue No. 34558920ML, Calbiochem, San Diego, CA, USA) to preserve the fluorescence. Cultures were subsequently studied under an Olympus AF-1000 confocal microscope (20× magnification), and at least 8 fields were analysed per sample, quantifying the total number of Ki67 or ssDNA positive cells divided by the total number of DAPI positive nuclei (proliferation or apoptosis ratio), in addition to Ki67, ssDNA, and MBP recording the optical density of each fluorophore (optical density arbitrary units) using the NIH IMAGEJ software version 1.53t for the analysis of the mean gray value, using the ROI manager in the images acquired by confocal.

### 2.3. Western Blot

The following is a brief explanation of the methodology used. Firstly, a culture of HOG (Human Oligodendroglioma Cell Line) cells was carried out, supplemented with the corticosteroid Methylprednisolone (Laboratorios Normon, Tres Cantos, Spain, SA) at different concentrations (0, 0.5, 5, 30, and 50 µM) and times (3, 5, and 10 days). Cell culture proteins were extracted with RIPA buffer (Thermo, Waltham, MA, USA) under aseptic conditions. Proteins extracted from HOG cells were quantified using the Bradford technique with Coomassie Plus, followed by Western blotting. MBP antibodies (Abcam, catalogue No. AB65988) were applied to the membranes to label an abundant protein present in the myelin of the central nervous system (CNS) and ß-Actin was applied as a control protein, followed by secondary antibody Li-COR 633. Finally, the membranes were revealed using the Odyssey CLx infrared imaging system (LI-COR^®^ 9140), and the images were analysed using Image Studio Ver 5.2 software.

### 2.4. Statistical Analysis

Each study and quantification were double-blinded. For parametric variables, we conducted an analysis of variance (ANOVA) with Tukey’s post hoc test. Statistical analysis was conducted with the SPSS software, version 20.0 for Mac (SPSS Inc.; Chicago, IL, USA).

## 3. Results

### 3.1. Methylprednisolone Reduces the Proliferation of HOG Cells by Reducing Their Cell Viability in a Dose-Dependent Manner

**Figure 2:**
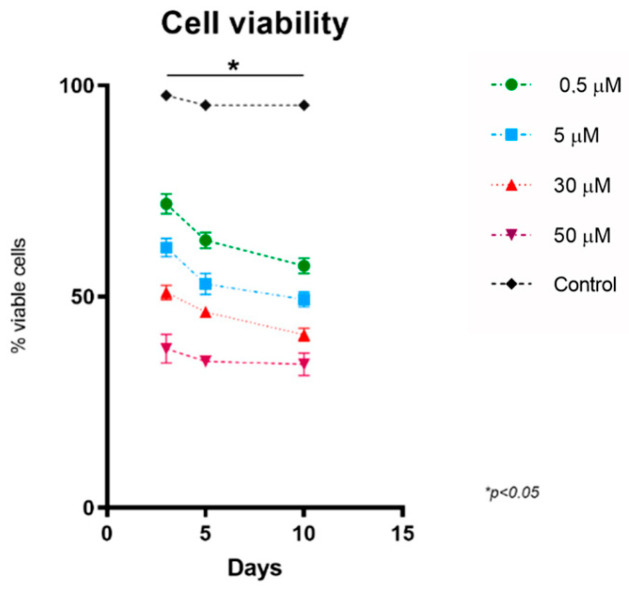
Cell viability of cell cultures. Any doses of methylprednisolone significantly reduced cells’ viability, with constant values at concentrations of 30 and 50 μM. N value = four.

**Figure 3:**
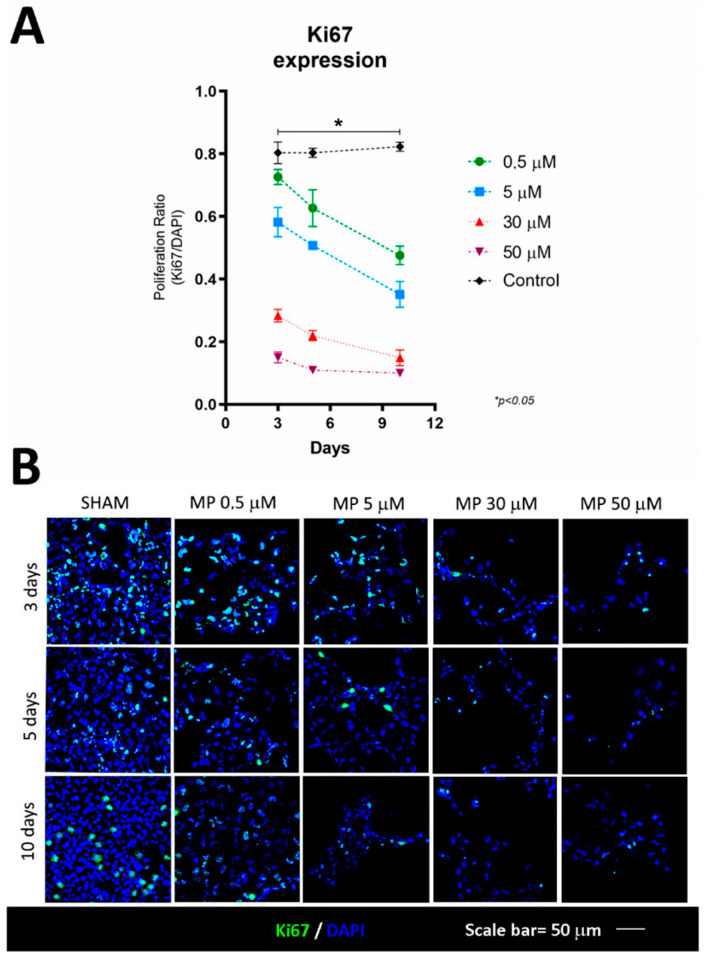
Evaluation of cell proliferation. (**A**) Ki67 expression decreased with respect to baseline; at 10 days, values were similar for all methylprednisolone doses, with significant differences compared to baseline. (**B**) Confocal microscopy images of Ki67 expression, showing lower cell proliferation at higher methylprednisolone concentrations and longer incubation times. N value = four.

Figure 2 shows how higher doses of methylprednisolone were associated with greater reductions in the cell viability of HOG cells, demonstrating that this drug decreases cells’ capacity to proliferate (F [2,8] = 8.571; *p* = 0.010) (Table 1). This effect was also reflected in the reduced expression of Ki67, which was also dose-dependent, and was observed from the earliest stages of the culture (Figure 3) (F [4,10] = 4.119; *p* = 0.031), with a decrease in the proliferative capacity of HOG cells (Table 2).

### 3.2. Methylprednisolone Induces Oligodendrocyte Cell Death in a Dose-Dependent Manner

**Figure 4:**
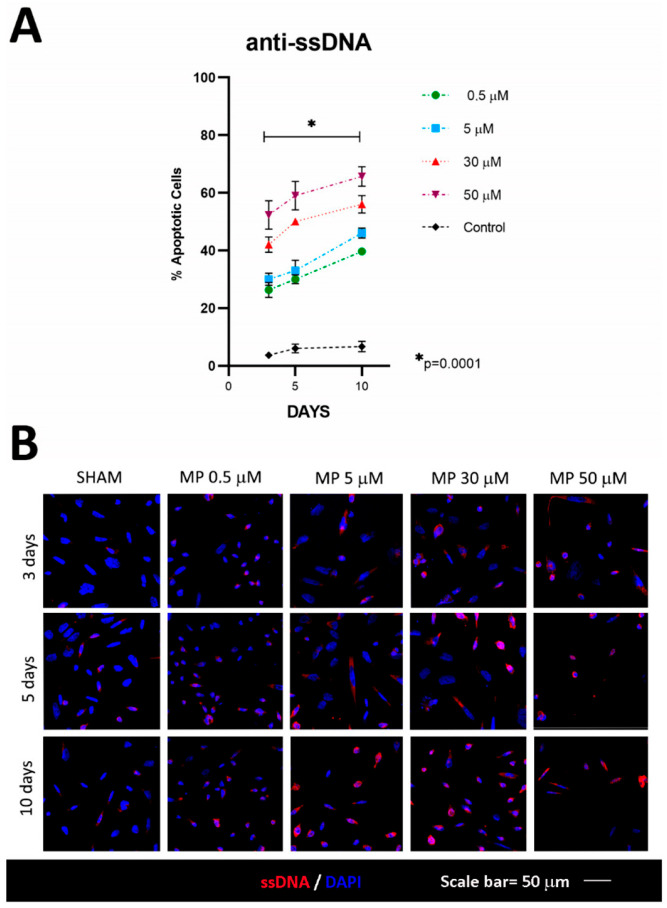
Evaluation of cell apoptosis. (**A**) Increased expression of ssDNA with respect to baseline; at 10 days, values were similar for all methylprednisolone doses, with significant differences compared to baseline. (**B**) Confocal microscopy images of ssDNA expression, showing apoptotic cells at higher methylprednisolone concentrations and longer incubation times. N value = four.

In the analysis of cell death via ssDNA labelling, it is shown how higher doses of methylprednisolone are associated with a greater increase in the death of HOG cells, the data being inversely proportional to those observed in the expression of KI67, demonstrating that methylprednisone affects cell viability. The differences were statistically significant at any concentration in reference to its comparative sham (F = 28.74; R square = 0.92, *p* = 0.001) (Figure 4A,B).

### 3.3. Methylprednisolone Reduces Oligodendrocyte Differentiation in a Dose-Dependent Manner

**Figure 5:**
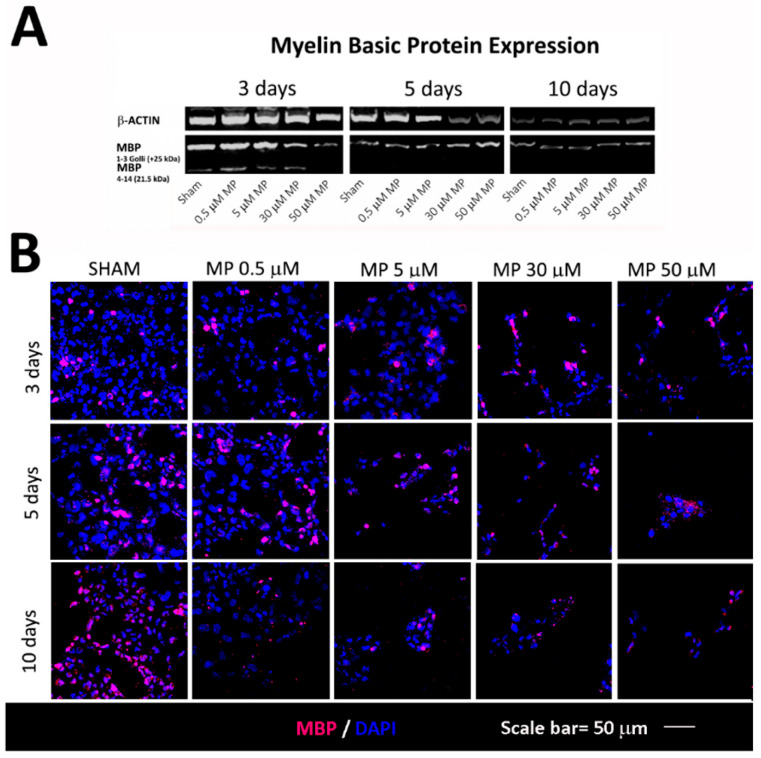
Expression of markers related with oligodendrocyte maturation. (**A**) Methylprednisolone administration was associated with a sharp reduction in MBP in 5 and 10 days, with a statistically significant difference compared to baseline. (**B**) Confocal microscopy images showing how methylprednisolone administration inhibits the expression of MBP, a protein closely linked to remyelination. N value = four.

Figure 5 shows how methylprednisolone is associated with lower levels of the marker of mature oligodendrocytes (MBP, closely associated with the phenotype of myelin-forming oligodendrocytes), suggesting reduced differentiation. In the WB analysis, a marked reduction in the MBP expression was linked to time and the concentration of methylprednisolone. It is noteworthy that in the samples of the sham group, and at low concentrations of methylprednisolone and in a short time, two bands related to MBP are observed; one is linked to oligodendroglial maturation (MPB 1–3 Golli 25 kDa), which remains in constant reduction with time, and the second band is related to MBP (4–14 21.5 kDa), which is closely related to remyelination (Figure 5A) (Supplementary Figure S1). This effect was dose-dependent and was observed from the earliest stages of the culture (F [4,10] = 16.24; *p* = 0.0002) (Table 3).

In addition, we performed other markers to check the identity of the cells after treatment. Among the markers used, PDGFR-a is one of those considered classic for the oligodendroglial lineage, showing that it was still expressed but with a different pattern from that observed in the sham group, and was more pronounced at a higher concentration of methylprednisolone (Supplementary Figure S2).

## 4. Discussion

While corticosteroids are known to delay myelination in the foetal brain [29,30,31], their effect in adults is less understood, although it has been suggested that they may have negative effects in stroke [32]. Due to their anti-inflammatory effect [33], corticosteroids are used to treat patients with MS, especially during relapses [34,35,36,37]. Independently of their anti-inflammatory effect, their influence on remyelination capacity in these patients is a controversial subject, with the limited number of studies performed reporting contradictory findings. In a study using a mouse model of lysolecithin-induced demyelination, Pavelko et al. [38] suggest that corticosteroids may promote remyelination. However, while that study suggests that the anti-inflammatory effect accelerates remyelination and repair, it does not address the question of whether this effect may in fact be explained by a reduction in demyelination, rather than due to the drug promoting remyelination. Triarhou and Herndon [39] also used a lysolecithin-induced demyelination model, finding that corticosteroid administration was associated with reduced remyelination. As the model involves local toxic demyelination, inflammation is less intense than in the experimental autoimmune encephalitis model; furthermore, while it enables easier analysis of remyelination [40] and has been used in research on potential remyelinating drugs [41], it presents the limitation that the pathogenic mechanism is not directly related to MS. A third study by Chari et al. [42] used a different model of toxic demyelination, injecting ethidium bromide into the spinal cord of rats. These authors observed reduced remyelination at one month compared to the controls, with a recovery at two months. They also found that corticosteroids did not affect the repopulation of OPCs but did influence their differentiation into oligodendrocytes; they also did not observe an increase in glial fibrillary acidic protein expression, suggesting that the drugs did not promote astrocyte differentiation. The authors consider that remyelination at 2 months is essentially explained by a reduction in the number of macrophages and in levels of inflammatory factors, rather than an increase in the capacity of OPCs to differentiate into myelin-forming oligodendrocytes. Alonso et al. [43] report that the prolonged administration of corticosteroids inhibits OPC proliferation, stressing that the possible benefits are influenced by inflammation, and that in the absence of inflammation, the drug would probably reduce the body’s remyelination capacity. Clarner et al. [44] used a toxic cuprizone-induced demyelination model, confirming that dexamethasone reduces remyelination in the corpus callosum by modifying the temporal pattern of OPC differentiation. This study includes data from in vitro experiments with cultures of oligodendrocytes (whose differentiation schedule is preserved) treated with hydrogen peroxide in order to analyse the consequences of the differentiation after the administration of corticosteroids, observing delayed remyelination. Halfpenny et al. [45] showed that corticosteroids inhibit the proliferation of precursor cells in a rat model of the CG4 cell line, with a capacity to differentiate into either astrocytes or oligodendrocytes. Finally, Chesik and De Keyser [46] found that corticosteroids acting on astrocytes downregulated oligodendrocyte differentiation.

In this study, we used a culture of HOG cells, which are human oligodendrocyte lineage precursor cells from an oligodendroglioma line. Previous studies by our group and other researchers have shown that these cells behave similarly to OPCs, expressing markers of precursor cells and of the oligodendrocyte lineage [47]. Given their ability to proliferate and differentiate to express PLP, MBP, and MOG and to generate myelin [48,49], these cells have been used in research on the mechanisms of myelination [48,49,50,51,52] and the influence of the immune system over OPCs [53,54,55]. Therefore, we considered them a good in vitro model to analyse the impact of corticosteroid treatment.

The addition of methylprednisolone to cultures of HOG cells causes a dose-dependent decrease in their proliferation and differentiation into oligodendrocytes, and also impairs the survival of these cells. Our results with human cells replicate the findings reported in studies with rodents, in a model that does not involve an inflammatory mechanism with potential to influence the results.

Undoubtedly, an interesting result is the analysis of apoptosis in HOG cell cultures with treatment of different doses of methylprednisolone, which our data found with the reports in the study by Diem et al., where in an EAE model, it is observed that the administration of methylprednisolone shows an exacerbation of apoptosis in an animal model that especially reflects the neurodegenerative aspects of MS. This paper provides evidence that there may be subgroups of patients with chronic autoimmune inflammatory disease of the CNS who are at risk of unwanted side effects of high-dose methylprednisolone therapy that could promote ongoing neuronal degeneration. These results suggest that combination therapies targeting the inflammatory and neurodegenerative aspects of MS should be developed in the future [56].

Several mechanisms are clearly involved in the drug’s effect on remyelination. The first possible mechanism is inflammation [57,58,59,60,61], which is associated with the role of microglia in the differentiation of OPCs [62,63]. The secretion of inflammatory factors, which, depending on the scenario, has a bimodal effect that either favours or impairs the differentiation of oligodendrocytes [64,65,66,67], may explain why the effect on myelination can change in acute situations, with the anti-inflammatory effect of corticosteroids potentially playing a role. Another mechanism is the action of corticosteroids on astrocytes, which may have a secondary effect on remyelination [68]; genetic mutations affecting astrocytes are known to affect myelination [69,70]. Oligodendrocyte lineage cells present corticosteroid receptors, whose expression varies according to the subtype of OPC. However, insufficient data are available to determine whether these receptors mediate the effect of corticosteroids on myelination. The OPC population is heterogeneous and specific to particular brain regions, with remyelination efficiency depending on the origin of the OPCs [71]. An analysis of OPCs from different brain regions may reveal the differences in the functional status [72], which may be influenced by their origin, location, or the presence of demyelination [12]. For instance, the grey and the white matter present different rates of OPC proliferation and differentiation into oligodendrocytes [73,74]. The understanding of these differences between regions is incomplete, with the further limitation that data from studies of rodents cannot be fully extrapolated to humans on account of the significant differences between rodent and human OPCs and oligodendrocytes [75]. Therefore, the differences between the OPC subtypes in the expression of these receptors [76] act as a confounding variable in analyses of their role in remyelination.

HOG cells present very similar characteristics to endogenous OPCs, but originate from a tumour; therefore, while they are useful in experimental studies, they cannot be used as implantable cells. However, data from studies using these cells may be extrapolated. Cell therapy with OPCs is a very promising approach, if the cells administered are able to repair myelin. Their administration, either alone or with biomaterials [77,78], may be useful, but it is essential to determine which factors may influence cells after implantation. In a previous study, we demonstrated that oligodendrocyte lineage cells administered by the intranasal route are able to access the brain and to migrate [47].

Although it is not the objective of the present work, our data could be useful to understand the metabolism, and therefore, the treatment of oligodendrogliomas, which are a rare subtype of diffuse gliomas, which represent <5% of all primary brain tumours and <20% of diffuse gliomas. Their treatment is complex and sometimes difficult because they turn out to be gliomas due to their heterogeneous topographic distribution and metabolism [79,80]. Recently, Batchu et al. provided a comprehensive description of oligodendrocyte heterogeneity; their data provide the basis for identifying new specific metabolic pathways for the treatment of location-defined oligodendrogliomas. This would expand therapeutic options and our understanding of its underlying pathobiology. Likewise, the development of histological markers for the detection of metabolic heterogeneity in steroid metabolism and oxidative phosphorylation would further facilitate therapeutic treatment [81,82].

In conclusion, protocols for the administration of oligodendrocyte lineage cells aimed at repopulating oligodendroglial niches or repairing demyelinated axons should not include corticosteroid treatment. Given the evidence that the effects of these drugs at high doses can undermine the objectives of cell transplantation, it is evident that the administration of methylprednisolone directly influences the death of oligodendroglial cells, in addition to inducing dose-dependent apoptosis.

## Figures and Tables

**Table 1:** Cell viability.

	Control	MP 0.5 µM	MP 5 µM	MP 30 µM	MP 50 µM	Day
**Mean**	97.67	72	61.67	51	37.67	**3**
SEM	0.3333	2.309	2.186	1.732	3.383	
**Mean**	95.33	63.33	53	46.33	34.67	**5**
SEM	1.202	1.856	2.517	0.8819	0.8819	
**Mean**	95.33	57.33	49.33	41	30	**10**
SEM	0.3333	1.856	1.764	1.528	2.646	

MP: methylprednisolone; SEM: standard error of the mean.

**Table 2:** Proliferative activity/Ki67 expression.

	Control	MP 0.5 µM	MP 5 µM	MP 30 µM	MP 50 µM	Day
**Mean**	2.285	2.172	2.021	1.125	0.6031	**3**
SEM	0.026	0.0142	0.099	0.075	0.0322	
**Mean**	2.378	1.466	0.838	0.408	0.268	**5**
SEM	0.036	0.061	0.095	0.014	0.065	
**Mean**	2.082	0.423	0.245	0.233	0.173	**10**
SEM	0.007	0.006	0.011	0.024	0.0211	

MP: methylprednisolone; SEM: standard error of the mean.

**Table 3:** Oligodendroglial maturation/MBP expression.

	Control	MP 0.5 µM	MP 5 µM	MP 30 µM	MP 50 µM	Day
**Mean**	0.248	0.2023	0.141	0.0457	0.01243	**3**
SEM	0.0124	0.0129	0.00406	0.00523	0.00202	
**Mean**	0.287	0.182	0.054	0.0012	0.00458	**5**
SEM	0.00427	0.00518	0.0865	0.002	0.000721	
**Mean**	0.3432	0.0601	0.0345	0.00259	0.00172	**10**
SEM	0.00723	0.00246	0.0038	0.00053	0.0024	

MP: methylprednisolone; SEM: standard error of the mean.

## Data Availability

Not applicable.

## Supplementary Materials

The following supporting information can be downloaded at https://www.mdpi.com/article/10.3390/cells12111515/s1, Table S1: Reagents and materials used in cell culture. Figure S1. WB data Figure S2: confocal image of PGFRα 50 μm.
