# Supplementary material for: In Vitro Effects of Methylprednisolone over Oligodendroglial Cells: Foresight to Future Cell Therapies
Source: Cells. 2023 May 30;12(11):1515. doi: 10.3390/cells12111515 (PMC10252523; doi:10.3390/cells12111515)
Supplement: Supplementary file 1 [file cells-12-01515-s001.zip › cells-2364621-supplementary.pdf]

Table S1

Reagents and materials used in cell culture

Reagent/Material	*Commercial Company*	*Reference*
Methylprednisolone	Sigma-Aldrich	M1750000
PBS	Gibco	10270-106
TrypLETM express	Gibco	12604-013
DMEM	Gibco	11320-074
FBS	Gibco	10270-106
Flask T25	Eppendorf	0030 710.126
Falcon 15ml centrifuge tube	Thermo Fisher Scientific	339650
5 and 10 ml pipettes	Fisherbrand	13-676-10J
trypan blue	Sigma-Aldrich	72-57-1

supplementary Figure S1

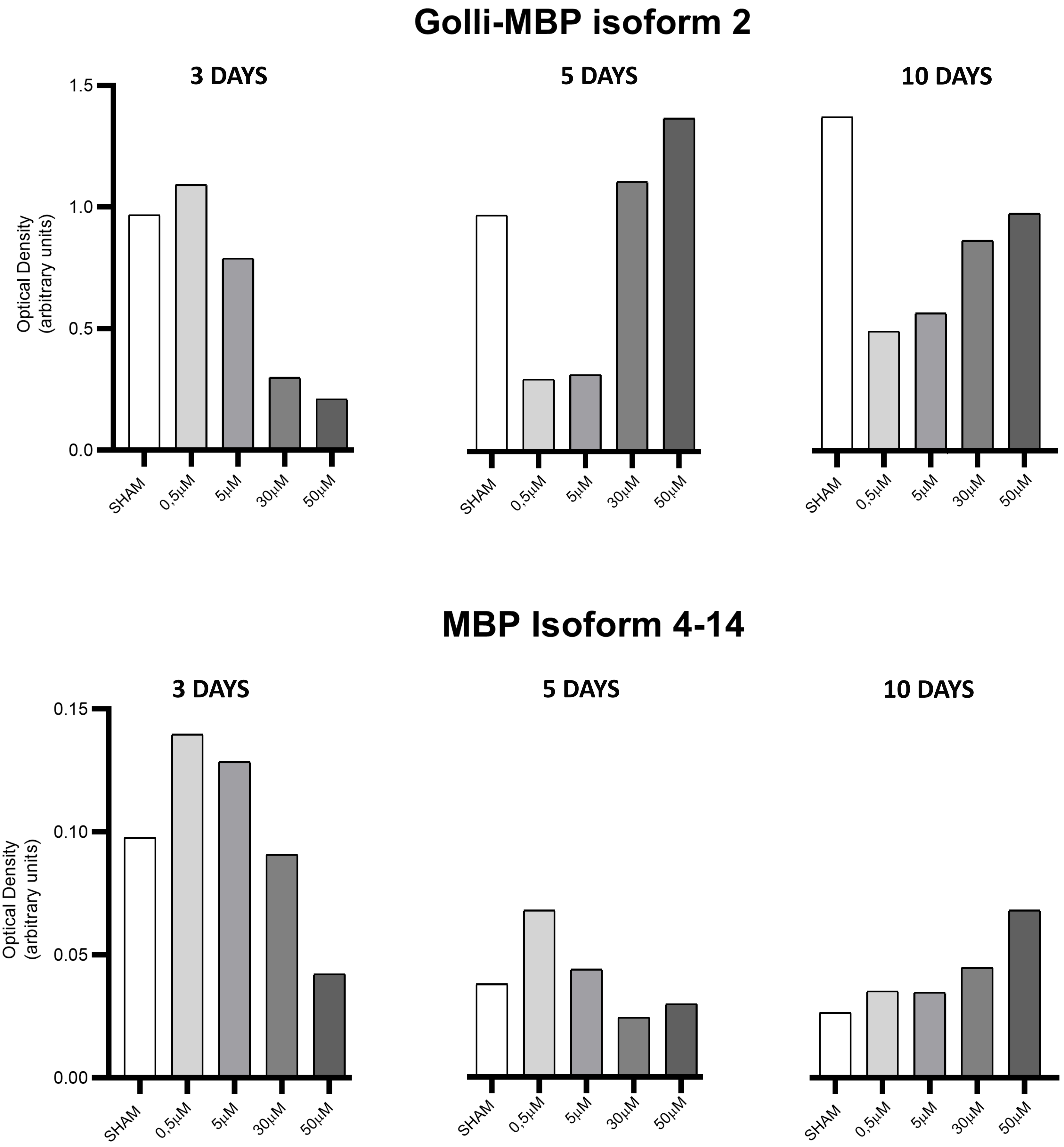

Supplementary Figure S2

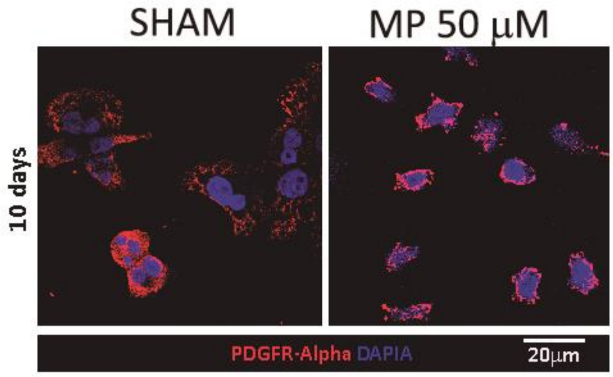

Example of image shows that the specific markers of oligodendroglial progenitors are maintained after corticosteroid administration (10 days), but with a different pattern.
